# Could the R2C2 Feedback and Coaching Model Enhance Feedback Literacy Behaviors: A Qualitative Study Exploring Learner-Preceptor Feedback Conversations

**DOI:** 10.5334/pme.1368

**Published:** 2025-01-17

**Authors:** Subha Ramani, Heather Armson, Tessa Hanmore, Rachelle Lee-Krueger, Karen D. Könings, Amanda Roze des Ordons, Marygrace Zetkulic, Joan Sargeant, Jocelyn M. Lockyer

**Affiliations:** 1 Harvard Medical School, MGH Institute for Health Professions Education, Boston, Massachusetts, US; 2 External Faculty, School of Health Professions Education, Maastricht University, NL; 3 Department of Family Medicine, Office of Continuing Medical Education and Professional Development, Cumming School of Medicine, University of Calgary, Calgary, Alberta, Canada; 4 Departments of Ophthalmology, Psychiatry, and Physical Medicine and Rehabilitation, Queen’s University, 99 University Ave, Kingston, Ontario, K7L 3N6, Canada; 5 Office of Continuing Medical Education and Professional Development, Cumming School of Medicine, University of Calgary, Calgary, Alberta, Canada; 6 School of Health Professions Education, Maastricht University, Maastricht, NL; 7 Departments of Critical Care Medicine, Anesthesiology, and Oncology, Cumming School of Medicine, University of Calgary, Foothills Medical Centre, 3260 Hospital Dr NW, Calgary AB, T2N 4Z6, Canada; 8 Hackensack Meridian School of Medicine, Nutley, New Jersey, US; 9 Continuing Professional Development and Medical Education, Faculty of Medicine, Dalhousie University, Halifax, Nova Scotia, B3H 4R2, Canada; 10 Department of Community Health Sciences, Cumming School of Medicine, 3280 Hospital Drive NW University of Calgary, Calgary, Alberta, T2N 4Z6, Canada

## Abstract

**Introduction:**

Feedback literacy (FBL) is a critical skill for learners encompassing four behaviors: appreciating feedback, making judgements, managing affect, and taking action. Little guidance has been available for clinical preceptors to promote FBL. The R2C2 feedback and coaching model that guides teachers through building Relationships, exploring Reactions and Reflections, discussing Content and Coaching to co-develop an action plan for follow-up may support FBL. This study sought to identify whether R2C2 conversations operationalized FBL behaviors and the factors that appeared to influence FBL.

**Methods:**

Based on data from a multi-institutional, qualitative study involving 15 dyads of learners (residents and medical students) and their physician preceptors, a secondary analysis of R2C2-guided feedback conversations and debriefing interviews was undertaken. A framework analysis mapped the data to FBL behaviors and explored factors that impacted behaviors in the context of the research and theories underpinning R2C2 and FBL.

**Results:**

Most elements of FBL behaviors were demonstrated in R2C2 conversations. Appreciating feedback and making judgements were most consistently noted. There was less evidence of managing affect as learners indicated acceptance of feedback. There was variability in the co-creation of action plans. Some created action plans, others had incomplete or no plan for immediate action or follow-up. FBL appeared to be impacted by learner-preceptor relationships, active learner engagement in feedback discussions, and personal characteristics.

**Discussion:**

Our analysis demonstrated that effective use of the R2C2 model could enhance FBL behaviors provided attention was paid to optimizing all phases of R2C2, particularly co-creation of action plans for follow-up.

## Introduction

Our understanding and study of feedback and coaching continues to evolve. Once viewed as a unidirectional communication from preceptor to learner, feedback has been reconceptualized as “a dynamic and co-constructive interaction in the context of a safe and mutually respectful relationship for the purpose of challenging a learner’s (and educator’s) ways of thinking, acting or being to support growth” [1]. Coaching is now considered a critical component of the feedback interaction and emphasizes feedback use and learner growth [2,3]. Various models have been developed to enhance feedback interactions and use such as the R2C2 Feedback and Coaching Model, Feedback Sandwich, and Pendleton Model among others [4,5]. Of these, the R2C2 model specifically integrates and emphasizes coaching. It is evidence and theory informed with four iterative phases in which the preceptor builds a *Relationship* with the learner (first R), explores their *Reactions and Reflections* (second R), confirms *Content* to be addressed (first C) and explicitly *Coaches for change* (second C) and engages the learner in co-creating an action plan [6,7,8]. The acronym R2C2 is derived from Relationship, Reactions, Content and Coaching. While feedback models offer guidance on preceptor behaviors and how to structure feedback conversations, they provide less insight into learner acceptance and processing of feedback, essential for effective feedback conversations [1,9,10]. Feedback literacy (FBL) has been identified as an essential skill in facilitating learner use and integration of feedback into their practice [11,12,13]. Clinical preceptors are well situated to support FBL as they have a longitudinal working relationship with learners and are able to provide ongoing feedback for learner growth and development, particularly in competency-based environments.

### Feedback Literacy

Feedback literacy (FBL) is defined as “the understandings, capacities and dispositions needed to make sense of information and use it to enhance work or learning strategies” [11]. FBL is considered critical to achieve maximum benefit from feedback, namely behavior change and professional growth, and benefits learners as well as teachers [14]. It includes four key behaviors: appreciating feedback, making judgements, managing affect, and taking action. For learners, it requires that learners understand what feedback is, have the capacity and motivation to use it productively, and appreciate both their own and the preceptor’s roles in these processes [11,12]. Foundational to FBL is the role of informed self-assessment [13]). For teachers, understanding the concept and phases of FBL could help facilitate action plan-centered feedback conversations with learners [14,15,16]. Feedback literacy principles emerged from social constructivist theory, which recognizes that shared and individual interpretations of feedback are developed through dialogue, sense-making and co-construction between participants. The goal is for learners to appreciate feedback messages, develop their capacities to make judgements about performance data and adjust their own learning and practice [11,12,13]. While FBL training for learners is being implemented for medical students and residents at some institutions ([12,17,18]), FBL for teachers has received little attention [14,19].

### R2C2 Model of Feedback and Coaching

The R2C2 model and its phases are underpinned by three theoretical foundations: informed self-assessment, humanism, and the science of behavior change [8]. Together the theories aim to foster learner reflection and self-assessment and engage learners as active participants in processing feedback and using it to develop plans for improvement. The R2C2 model has been studied extensively in varied clinical settings and disciplines [6,7,8,9,20,21,22]. Important outcomes demonstrated include: fostering self-assessment and reflection [23]; coaching content and process [9]; emotional reactions [8]; and supervisor-learner relationships [7,8,9]. Generally, the focus for use of the R2C2 model has been upon preceptors, and learners have been infrequently engaged in preparation.

There are epistemological similarities between R2C2 and FBL, related to their sociocultural and social constructivist foundations. The main focus of each may differ in some ways, with R2C2 guiding preceptor behavior and structure of the feedback conversation and FBL shaping learners’ uptake of feedback and implementation of action plans. However, both aim to promote learners’ engagement through reflection and self-critique, which can then guide action plans. Both R2C2 and FBL acknowledge the need for collaboration and an active role for each participant in the conversation [7,11,12]. While evidence informed practical strategies to inform FBL have been described in undergraduate and postgraduate medical education [15,17], there is little known about the feedback approaches and tools that preceptors use that may enhance feedback literacy [14], particularly in clinical settings. With a few exceptions [15,17,18,19], most of the FBL work has focused on non-clinical settings [11,15,24].

This study sought to identify whether the R2C2 feedback and coaching model as applied by preceptors within clinical settings could encourage learners to operationalize the four core behaviors of FBL (appreciating feedback, making judgements, managing affect, and taking action) along with the factors that appeared to influence FBL behaviors in R2C2 preceptor/learner feedback discussions.

## Methods

### Study Design

We conducted a secondary analysis drawing on data collected for a prior study to examine the utility of the R2C2 model for in-the-moment feedback [20,25]. Relevant to our study purpose, secondary analytic strategies enabled us to conduct an exploration of learner FBL behaviors within the context of R2C2 guided feedback conversations, an aspect we had not previously considered.

### Data Collection

Data from 15 dyads of learners and preceptors working in clinical settings were collected, as described in our previous study [20]. Faculty and learners from five academic institutions in the US and Canada participated in the study. There were 13 preceptors: six were internal medicine physicians; two were physiatrists; and one each from dermatology, geriatric psychiatry, rheumatology, palliative care medicine and ophthalmology. Two preceptors were part of two dyads. All but two learners were postgraduate trainees (two clinical clerks, four PGY-1, five PGY-2, one PGY-3, two PGY-4 and one PGY-5). We selected the continuum of learners in clinical settings from undergraduate through residency to obtain a breadth of perspectives. We purposely selected data from our in-the-moment study which showed that the R2C2 model could be adapted successfully for use in formative feedback discussions occurring shortly after observation of a clinical encounter. Both learners and preceptors were trained in the R2C2 approach for the prior study, thus we examined FBL skills among learners familiar with R2C2. Each dyad engaged in two feedback sessions that applied the R2C2 model, followed by individual interviewer-led debriefs with the preceptors and the learners within two days of the feedback session, and a second debrief with the learner two weeks later. A researcher at each site provided R2C2 training to participants at their respective institutions and instructed them on the process. Learners recorded the conversations and uploaded them to a secure folder accessible to investigators only; a team member from a different institution conducted and recorded all debriefing conversations. Transcripts were de-identified prior to analysis by the entire team.

### Data Analysis

The team drew on the original data, each set comprising two recorded feedback discussions between preceptor and learner and a de-brief interview each with the learner and the preceptor, to carefully examine and conduct a secondary analysis [25] through the lens of FBL behaviors [11,16,17,18,26]. Secondary data analysis (SDA) is a “respected, common, and cost-effective approach to maximizing the usefulness of collected data” [27]. Four approaches to SDA include: a different unit of analysis than the parent study; in-depth analysis using a subset of data; analyses of important data not sufficiently focused on earlier; and analyses that combines original and newly-collected data and refines the parent study’s purpose [27]. This study focused on a different unit of analysis than the previous study and adhered to all conditions of ethics approval which was sought to explore different aspects of learner-preceptor feedback conversations, guided by the R2C2 model and conducted by the same research team. Following review of FBL literature, several rounds of discussion through meetings and e-mails, the team agreed that some of the learner FBL behaviors, namely, appreciating feedback, making judgements, managing affect and taking action, were present within our data. We reviewed and slightly modified the descriptors of each of the four phases for clarity of analysis as a research team, shown in Table 1. While recognizing that a more nuanced approach could be taken to this analysis [12], through multiple group discussions, we selected the descriptors believed to be most applicable to the clinical context and the application of the R2C2 model in-the-moment. These descriptors were formatted into a coding template which each member of the team applied to one or more complete sets of dyad data to examine how the R2C2 model might align with the core behaviors of FBL (see appendix for supplemental coding form). For this study, we focused on broad FBL behaviors rather than the microskills as this was a preliminary exploration into the overlap and intersection between the two models.

**Table 1:** Descriptors for feedback literacy behaviors.


FEEDBACK LITERACY CATEGORY	DEFINITION

Appreciating feedback	Demonstrates understanding and values the role of feedback in improving performance and their active role in these processes.

Making judgements	Demonstrates capacity for critical reflection and self-assessment and has insight into strengths and areas for further development.

Managing affect	Maintains emotional equilibrium and avoids defensiveness when receiving critical feedback.

Taking action	Engages in conversations about steps required to integrate feedback to enhance performance including consideration of strategies and performance. Provides evidence of implementing agreed upon plans.


We used framework analysis [12,28,29] to understand the R2C2-in-the-moment (itM) data vis a vis the broad behaviors of FBL. All authors were involved in the parent study and participated in the coding, analysis and writing. After each member independently applied the coding template to two or more dyad datasets, we finalized the coding template after group discussions. We then assigned each of the dyad data to a pair of research team members. Each researcher independently examined one to three complete sets of dyad transcripts and used the coding template (Appendix 1) to document examples of how the stages of the R2C2 model were applied (e.g., key phrases) along with researcher perceptions on whether, how and what FBL behaviors were demonstrated, and factors that seemed to influence how FBL was expressed. As a group, we created an Excel spreadsheet summary that captured the alignment between R2C2 data and FBL behaviors. We identified illustrative quotations from the dyads which demonstrated the alignment. Finally, drawing on the components, theories and research underpinning R2C2 and FBL and our discussions, we examined how our dyads demonstrated FBL and how R2C2 might be a useful way of supporting FBL.

### Rigor and Quality

All team members had access to the transcripts, codes, summary sheets, and manuscript drafts. Through frequent virtual meetings between pairs of research team members as well as the full team, we shared data interpretations, resolved conflicts and reached consensus. We conceptualized specific aspects of the study, challenging assumptions and theories to capture the major ideas as our thinking evolved in an iterative way. Our approach was consistent with our previous work using the in-the-moment feedback data [6].

The research team included four PhD educators (JML, JS, KDK)); two physicians with graduate degrees in education (HA, ARdO), one physician (SR) and one researcher (RLK) with a PhD in health professions education, one physician extensively engaged in R2C2 research and training (MGZ) and one researcher with a Master’s in education (TH). All investigators were trained in qualitative research methodology, and none were in a position of power over participants. Through frequent team meetings, we also reflected on our positions as clinician or non-clinician researchers, and discussed how involvement in our previous research on in-the-moment feedback may have influenced our interpretations.

## Results

The original R2C2 – in the moment study included 15 dyads of learners (two undergraduate medical students, 13 residents) and 11 clinical preceptors. Below, we describe the four components of FBL and indicate how participant narratives from the R2C2 transcripts mapped to each of the FBL components along with representative quotes. The first letter refers to the site of data collection, L refers to learner and P to preceptor.

### Appreciating feedback

Appreciating feedback category of FBL was defined as learners’ valuing the impact of feedback in improving performance and their own active role in these processes. The appreciation was most evident when the learner identified clear goals and communicated to their preceptor what they wanted observed. Goal setting appeared to occur more readily when the preceptor initiated the conversation by asking the learner to identify performance areas or challenges as the focus of feedback. For learners whose relationship with the preceptor was longitudinal and feedback was a regular occurrence, the learner was able to refer to previous goals and action plans and view feedback as part of a developmental process. Learners emphasized that feedback was more helpful when they were directly observed, the feedback was concrete and occurred in real time.

I’ve noticed if I ask someone for some direct feedback, people are very willing to just sit and help. And I think that having even a few minutes one-on-one with someone, it goes a long way for me. So, it just felt like a perfect context to get some feedback and then get – go back out there, try to – try to practice it and try to learn and try to get better. (D.L4)

### Making judgements

Making judgements refers to learners’ demonstration of their capacity for critical self-reflection with insights into their strengths and areas for further development. Most learners in the study acknowledged the importance of reflecting on their work and appeared able to assess their own performance. The learners made their own judgements about the quality and utility of the feedback data received. They suggested that preceptors could facilitate this process by asking open-ended questions, allowing the learner to lead the session, and creating an environment in which it was safe to have an open and honest discussion.

One resident described a time when they were able to receive and sort through information then judge for themselves what areas they could improve.

This felt much more deliberate and that she (preceptor) had clearly paid a lot of attention to the conversation and was drawing out details about the interaction and sort of getting to reflect on those together. And so, it definitely felt of high value to me from a feedback perspective, and certainly felt like I was receiving individualized mentoring. (A.L1)

Learners also recognized that the onus was on them to sift through the feedback data they received and judge what was relevant to their established goals, and select specific areas for improvement that they would then carry forward into action plans.

…ultimately not all that information is relevant, and I need to learn to really decide at an earlier stage what I should be pursuing and what isn’t. (A.L2)

### Managing affect

Though most learners did not talk directly about addressing emotion, they talked about relationships which enabled them to avoid defensiveness and facilitated their acceptance of constructive feedback. Preceptors helped learners to manage affect by normalizing that all professionals have areas for improvements and shared concrete strategies to help learners handle their emotional responses.

…a lot of us in medicine have been overachievers and perfectionists and Type A people and we often struggle with receiving feedback that says we need to improve in an area. It’s almost seen as a failure. And so, I think really establishing that relationship properly initially shows that the goal of the person giving the feedback is not to say, “Hey, you’re doing this wrong.” But it is to say, “Hey, you’re doing a great job and you’re moving forward through this, there’s a few areas for improvement that we can work on.” (A.P2)

### Taking action

The “taking action” aspect of FBL requires learners to engage in or initiate strategies to integrate feedback and implement agreed upon plans. Some dyads were able to create specific action plans. In most cases, the preceptors directed the discussion towards action plans. Action plans were enabled when learners documented feedback, which facilitated implementation and recall. Collaboration between the learner and preceptor appeared to enable the development of an implementable action plan and encouraged learner agency.

And it was collaborative in that way and it seemed to kind of build and strengthen our bond and also helped I felt to frame the observation not so much as …someone’s watching you to criticize but rather…we’re in this together for you to be better and, you know, you’re going to achieve competency and this is the goal, this is the way that we achieve it. So yeah, it was actually quite good for promoting collaboration. (E.L3)

There were missed opportunities for preceptors to probe and guide the learner in developing a specific plan. While some dyads identified a future goal, others did not overtly discuss goals even if the resident had some ideas.

We didn’t really set goals for next time since it was going to be months until I did it anyway. I do know that there are things that I need to work on, but those have been smoldering in the back of my mind for a while. I haven’t officially recorded it. (D.L2)

### Factors impacting FBL behaviors

From our data, we identified three important factors that appeared to influence the expression of FBL behaviors: the preceptor-learner relationship; learner willingness to seek feedback; and preceptor ability to facilitate learner engagement.

Longitudinal relationships seemed to make it easier for learners to demonstrate FBL behaviors.

I feel like that is the part (relationship) that really feels ….more like a two-way street rather than just one-way…. I think that the other thing that made it feel collaborative was really that we, in some ways, have directly partnered to take care of these patients, that we’ve had time to build this relationship between each other over the course (D.L2)

Learners in our study were able to articulate their reflections, share reactions, and create an action plan that they and the preceptor would be able to monitor and follow. For the two learners who didn’t have a longitudinal relationship (e.g., were doing a short elective or off-service rotation), successful use of R2C2 benefited from both the preceptor and learner being more focused on ensuring that the learner’s goal for the observation was determined *a priori*. The preceptor had to ensure there was a fulsome discussion of the goals for performance observation, and a plan developed to carry the learner ahead to other rotations.

…having the preceptor lay out some concepts about what was going to be observed or what I’d be interested in having observed, and having me select a particular thing that I wanted to receive feedback on….sort of got me to think a little bit more about my own learning objective and just what I wanted to get feedback on (A. L1).

The feedback and debriefing conversations indicated that a few personal attributes could have played a role in demonstration of FBL behaviors. Learners who were more willing to reflect on areas for improvement were more able to engage in conversations, integrate feedback and consider and discuss strategies for improvement. They emphasized that open-ended questions from preceptors facilitated self-reflections and learner agency.

….open-ended questions around what I was looking to accomplish…helped stimulate more collaboration about what I should do in the future rather than it feeling simply prescriptive about what I should change, and more about allowing me to help drive that process (A.L1).

Preceptors dominating the conversation and reverting to a lecturing mode seemed to inhibit learner agency, engagement, and participation. Learners did not demonstrate characteristics of FBL behaviors when they had a passive role in the conversation. When learners had fewer opportunities to contribute to the conversation, it was not clear whether they appreciated the feedback, had the space to share emotional reactions, were able to develop implementable action plans, or develop a follow-up plan. Box 1 illustrates such an interaction in which the learner responds with ‘yeah’ and ‘ok’ offering little in the way of their own perceptions and the preceptor does not enable the learner to participate in a meaningful way in the discussion.

**Box 1 Learner lack of engagement when preceptors dominate the conversation**

Preceptor: You sort of gave her an answer rather than saying, “Tell me what worries you about surgery,” and then let her see. That often can be more—and she was very chatty, I have to say. I do think that sometimes you have to pull those people back in, but I did think that there were a few opportunities where you could say, “Tell me more,” or—and there was a point where she was telling us about going to the gym, and I was sort of curious to hear a little bit more about—she kept saying, “Well, I started and then I added five minutes,” but I kind of want to hear, well, what’s she doing now? How many minutes has she made it up to? It sounded like she was maybe sequentially—and then also, “Do you have any goals? What are your exercise goals? What are your weight loss goals?” She’s clearly making progress, and sometimes I don’t even push people for goals when they first start, but I think here, like she said, “I don’t want to have surgery. I want to do this myself,” and she’s clearly making progress with her dietary changes, although it’s tough, right? She was talking about how she really likes rice, and now she can only eat a little bit. Sometimes I’ll sort of say, “Tell me, do you have any goals? What are your goals for your weight?”

Resident: Yeah.

Preceptor: “What are your goals for your exercise?” If she doesn’t know, you could help her work through some.

Resident: Mm-hmm. That would be nice, I guess, just to have her set herself a benchmark which is more powerful than me setting her a benchmark.

Preceptor: Yeah, and sometimes you can hear what she has to say, and then help guide that benchmark too. The thing that I worry the most about her is that she’s gonna plateau and give up, so what I wanted to hear her tell us would be, “Well, I have a goal of exercising every day,” or, “I’m gonna go to the gym three times—” Actually, ideally I’d like her to say seven times a week, but, [laughter] if not, five times a week. You can also, even if her weight loss stops, you can see how is she doing with her exercise goal, and then could we make a new goal? Because I think this is the other thing people—I’m pretty impressed with how much weight she’s lost already, because I feel like, often, the heavier people get, the harder it is to even get started because they’re just so entrenched in the lifestyle that led to them being like that. I think if she’s really lost 14 pounds, good for her. That’s pretty impressive. I thought you did a nice job of encouraging that. You know, “You’re making good progress. Good for you. This is hard, but you’re committed,” and those kinds of things. I think that affirmations can be really helpful. I think what the challenge is gonna be next is, she’s gonna realize, “Oh, wait, it’s not as easy as it was,” and then the risk of getting frustrated, so then having not just weight goals, but physical activity goals and diet goals could be really helpful.

Resident: Like the intermediate steps to losing weight.

Preceptor: Exactly.

Resident: Okay.

[D.L2.P2]

## Discussion

This study provided a unique opportunity to explore whether R2C2, when carefully applied, might guide preceptors and learners in operationalizing the behaviors required for FBL. We found that appreciating feedback and making judgements were the most consistently applied aspects of FBL. There was variability in the co-creation of action plans, where some created specific action plans, others had incomplete or no plan for immediate action or follow-up. There was less evidence of managing affect in the dataset we analyzed. Our study also illuminates factors that may support or create barriers to FBL. There appear to be many points of intersection across the R2C2 and FBL behaviors and feedback training for teachers and learners could incorporate elements of both. Building relationships could enhance appreciation for feedback and managing emotion could foster self-reflection of strengths and areas for improvement. These, in turn, can guide how learners might consider numerous points of feedback before formulating specific action plans for improvement. Finally, feedback training needs to emphasize coaching for implementation of action plans for practice and behavior change. The coaching is a deficiency noted in our results.

R2C2 is iterative [6,30]. Preceptors can return to previous stages as needed to enhance learner engagement. This may explain why the FBL behaviors inherent in appreciating feedback and making judgements were more consistently demonstrated within the dyads. It is also not surprising that building a relationship could influence all FBL behaviors, namely appreciating feedback, making judgements, managing affect, and finally taking action. Exploring reactions and reflections are well aligned with making judgements about one’s performance and managing affect. Confirming content is fundamental in both FBL and R2C2 and required for learners to make judgements and decide on actionable areas to take forward. While learners in this study did not demonstrate overt emotional reactions to constructive feedback, learned behaviors related to controlling emotions in the clinical workplace, especially as front-line clinicians, may impede overt learner articulation of their reactions [31,32]. Co-creating action plans/taking action, have been identified in both the R2C2 literature [6,7,8] and FBL literature [24] as being more challenging to achieve consistently and with clarity.

Many factors can influence whether and how R2C2 could enhance learner FBL behaviors. Addressing these factors could help to design strategies to enhance FBL behaviors within R2C2 feedback and coaching conversations. Effective feedback requires relationship building and a recognition that this is a shared responsibility [8,14,20]. Ensuring that the learner has sufficient time to identify their goal for feedback and recount factors leading up to that goal will help set the agenda, even in short working relationships. Important personal characteristics include learner agency [17] and learner and preceptor commitment to authentic feedback [7,14]. Coaching is a higher order skill. Preceptors need time and practice to develop the skills necessary to guide, but not dominate, the coaching discussion [6,9,33]. As our results indicate, the more the preceptors dominate the feedback conversation, the less learners engage. In addition, preceptors need to train and empower clinical learners in developing and implementing action plans for growth and improvement. When working with clinical trainees who may be struggling in specific performance domains, extra attention may be needed to provide autonomy appropriately and engage them in effective self-calibration [16].

There are limitations to the study. This was a secondary analysis of data collected to demonstrate that the R2C2 model could be operationalized for in-the-moment feedback rather than in longitudinal progress meetings. The de-briefing interviews did not address FBL directly; thus, we cannot make sweeping conclusions about the impact of R2C2 on FBL behaviors. Learner statements about acceptance of feedback may not have fully reflected their emotional responses to constructive feedback. The study included preceptors and learners who were trained in R2C2 use, which could have influenced their approach to the feedback conversations. These findings, especially as they pertain to FBL behaviors, may not be directly applicable to a participant population without training, other healthcare disciplines, or different training contexts. Further, where preceptors and learners utilized R2C2 ineffectively, it was difficult to understand completely how trainees used the discussion to enhance their work or learning strategies.

**Figure 1:**
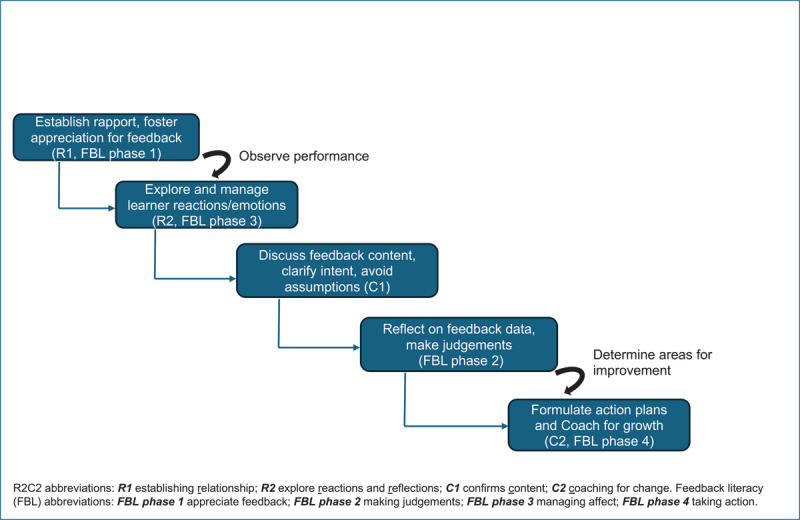
A proposed framework for bidirectional, learner-engaged feedback conversations integrating R2C2 and FBL behaviors. Intersection of R2C2 and Feedback Literacy Behaviors. In the R2C2 model, teachers actively facilitate learner engagement. In the FBL model, learners’ skills and behaviors are more central, but teachers need to actively promote learner behaviors. Knowledge and application of both models could lead to optimal engagement from both sides and behaviors that incorporate teacher and learner perspectives.

While our data indicate that the underpinning theoretical principles, particularly self-assessment [7,11,13], and individual phases/behaviors of R2C2 and FBL appear to be aligned, the impact of R2C2 on feedback acceptance and application, namely FBL, needs to be assessed directly among learners. Future training of both preceptors and learners in the R2C2 method could include explicit attention to FBL behaviors so that preceptors can pay more attention to recognizing and promoting learner FBL. Facilitating learner engagement in identifying and acting upon areas for improvement might promote more effective coaching (the second C in R2C2 and taking action in FBL) and enable effective changes in behavior and practice. Our study has some notable implications for other health professions educators. We could see a true intersection of two important feedback models using the following steps as depicted in Figure 1:

1. Establish rapport and foster appreciation for feedback (R1, FBL Phase 1)
2. After an observed clinical encounter, provide an opportunity to express and manage emotion followed by self-reflection on performance (R2 and FBL Phase 3)
3. Discuss content of observation, avoiding assumptions about behaviors (C1)
4. Encourage learners to reflect on feedback data, make judgements on application and select key areas for improvement (C1 and FBL Phase 2)
5. Formulate action plans for improvement and coach for growth (C2 and FBL Phase 4)

## Conclusion

There are many areas of alignment between the R2C2 model and FBL framework. Specifically, the first step of R2C2 (establishing relationships) appears to be critical in setting the tone for learners to actively engage in and demonstrate feedback literacy. If coaching principles are to be applied effectively for practice and behavior change, the last phase of both models needs to be emphasized, i.e., coaching for growth and co-creation of action plans for change (R2C2) and taking action (FBL) respectively. Effective application of the R2C2 model by preceptors could enhance FBL in the context of clinical work when careful attention is paid to promoting learner agency and engagement in the process.

## Additional File

The additional file for this article can be found as follows:

10.5334/pme.1368.s1 Appendix 1. Feedback Literacy “in the moment” and R2C2 codebook.
