# Supplementary material for: Could the R2C2 Feedback and Coaching Model Enhance Feedback Literacy Behaviors: A Qualitative Study Exploring Learner-Preceptor Feedback Conversations
Source: Perspect Med Educ. 2025 Jan 17;14(1):9–19. doi: 10.5334/pme.1368 (PMC11740720; doi:10.5334/pme.1368)
Supplement: Appendix 1. — Feedback Literacy “in the moment” and R2C2 codebook. [file pme-14-1-1368-s1.pdf]

Feedback Literacy "in the moment" and R2C2 codebook

Research goals:

- To explore whether the R2C2 framework facilitates feedback literacy among residents overall
- To examine what specific aspects of feedback literacy may be facilitated by R2C2

DYAD Number: **DR2P2.** **Reviewer:**

**Literacy category**	**Definition**	**Sample quotes from**
Appreciating feedback	Demonstrates understanding and values the role of feedback in improving performance and their active role in these processes.	
Making judgements	Demonstrates capacity for critical *reflection and self-assessment and has insight into strengths and areas for further development.	
Managing affect	Shows willingness to share emotional reactions as appropriate after clinical encounters with preceptor. This could indicate that a safe space has been established.	
Taking action	Engages in conversations about steps required to integrate feedback to enhance performance including consideration of strategies and performance.<br><br>Provides evidence of implementing agreed upon plans.	

***Note**: Reflection added after April 2023 team meeting to emphasize that reflection is central to informed self-assessment and hence to making accurate judgements, a finding from earlier self-assessment research and our ongoing R2C2 research.*

Commentary: Please comment on

1. alignment between R2C2 phases and the literacy category,
2. how 'literate' you thought the resident actually was,
3. anything else that can help address the 2 research goals, above.
4. (4) evidence of factors that seem to play into feedback literacy, or influence demonstration of feedback literacy:
    a. The more that preceptor talks, the less the learner engages
    b. Evidence that R2C2 is preceptor dependent
    c. Importance of training both preceptor and learner
    d. Relationship between the two
    e. Curiosity of learner
    f. Personal characteristics of both preceptor and learner
    g. Effective feedback only occurs when learner uses feedback and takes action
5. any other insights you have from reviewing the dyad that might be helpful in understanding FBL

Reference: *Carless & Boud (2018) The development of student feedback literacy: enabling uptake of feedback, Assessment & Evaluation in Higher Education, 43:8, 1315-1325, DOI: 10.1080/02602938.2018.1463354 To link to this article: https://doi.org/10.1080/02602938.2018.1463354*
